# Supplementary material for: Feasibility of using alternative swabs and storage solutions for paired SARS-CoV-2 detection and microbiome analysis in the hospital environment
Source: Microbiome. 2021 Jan 22;9:25. doi: 10.1186/s40168-020-00960-4 (PMC7821463; doi:10.1186/s40168-020-00960-4)
Supplement: Supplementary file 2 — Additional file 1: Supplemental Figure 1. Impacts of storage solution or swab type on RNA quality as measured by RNA Tapestation High Sensitivity kit. Supplemental Figure 2. Impacts of sample-type (eluent vs. swab head) on RNA recovery by swab used. Supplemental Figure 3. Adsorption volumes per swab type. Supplemental Figure 4. Total variation of human Rp gene detected from floor samples in limit of detection experiment. Supplemental Figure 5. Variation of human Rp gene amplification across swab types. Supplemental Figure 6. Intra-assay variability compared across positive controls, sample _types, and swab types. Supplemental Figure 7. Comparison of 16S rRNA microbial composition of paired patient and sample type samples collected using different swabs (in triplicate) using Unweighted UniFrac PCoA plot. [file 40168_2020_960_MOESM2_ESM.docx]

**Supplemental Figure 1. Impacts of storage solution or swab type on RNA quality as measured by RNA Tapestation High Sensitivity kit.**

a) All direct-swab extracted RNA grouped by storage buffer and swab type. b) Samples grouped by storage buffer, no significant difference in RNA integrity number (RIN) values between storage buffers (95% EtOH vs. 91% isopropanol) (Mann-Whitney). c) Swab extracts grouped by swab type only and compared to determine if swab type has an impact on RNA quality (Kruskal-Wallis test).

**Supplemental Figure 2. Impacts of sample-type (eluent vs. swab head) on RNA recovery by swab used.**

Comparison human RNA recovery across six swab types (SYN=synthetic ‘commercial’, BDF=BD foam ‘commercial’, TMI=BD TMI ‘commercial’, CGp=plastic ‘consumer-grade’, Pu=Puritan ‘commercial’, CGw=wood ‘consumer-grade’), **a)** extracted from 200 μL  eluent or **b)** swab head (Group comparison using Kruskal-Wallis)

**Supplemental Figure 3. Adsorption volumes per swab type.**

Samples tested for normality using Shapiro- Wilke test (not significant, P=0.12, W=0.8357).

**Supplemental Figure 4. Total variation of human Rp gene detected from floor samples in limit of detection experiment.** a) Variation of Rp across swab types and subsequent dilutions. b) total yield of human Rp across aggregated swab types.

**Supplemental Figure 5. Variation of human Rp gene amplification across swab types.**

(left) Surface swabs were collected from the floor of a laboratory to compare efficiency across swab types; consumer-grade cotton (CGp), The Microsetta Initiative cotton swabs (TMI), and synthetic (SYN). (right) PE surface swab samples were collected with the same protocol in the hospital. The variation across hospital samples is greater than the variation among swab types.

**Supplemental Figure 6. Intra-assay variability compared across positive controls, sample _types, and swab types.**

a) Coefficient of variance calculated across pairwise swabs taken within each room/patient replicate and then distributions compared for bedrail, floor, nasal swab, and nasal eluent samples along with positive controls. b) The CV was also grouped and compared by swab type.

**Supplemental Figure 7.** Comparison of 16S rRNA microbial composition of paired patient and sample type samples collected using different swabs (in triplicate) using Unweighted UniFrac PCoA plot. a) floor samples, b) nasal samples. Swab types: square = SYN, circle = CGp, star = TMI.
